# In Vitro and In Vivo Evaluation of Nanostructured Biphasic Calcium Phosphate in Granules and Putty Configurations

**DOI:** 10.3390/ijerph18020533

**Published:** 2021-01-11

**Authors:** Jhonathan R. B. Nascimento, Suelen C. Sartoretto, Adriana T. N. N. Alves, Carlos F. A. B. Mourão, Victor R. Martinez-Zelaya, Marcelo J. Uzeda, José M. Granjeiro, Pietro Montemezzi, Monica D. Calasans-Maia, José A. Calasans-Maia

**Affiliations:** 1 Graduate Program, Dentistry School, Universidade Federal Fluminense, Niteroi 24020-140, Brazil; jhonathan_bn@hotmail.com (J.R.B.N.); mouraocf@gmail.com (C.F.A.B.M.); 2 Oral Surgery Department, Dentistry School, Universidade Veiga de Almeida, Rio de Janeiro 20271-020, Brazil; susartoretto@hotmail.com; 3 Oral Surgery Department, Dentistry School, Universidade Iguaçu, Nova Iguaçu 26260-045, Brazil; mjuzeda@gmail.com; 4 Oral Diagnosis Department, Dentistry School, Universidade Federal Fluminense, Niteroi 24020-140, Brazil; aterezinhanovellino@gmail.com; 5 Brazilian Synchrotron Light Laboratory (LNLS), Brazilian Center for Research in Energy and Materials (CNPEM), Campinas 13083-100, Brazil; victor.martinez@lnls.br; 6 Oral Surgery Department, Universidade Federal Fluminense, Niteroi 24020-140, Brazil; monicacalasansmaia@gmail.com; 7 Directory of Life Sciences Applied Metrology, Instituto Nacional de Metrologia, Qualidade e Tecnologia (INMETRO), Duque de Caxias 25250-020, Brazil; jmgranjeiro@gmail.com; 8 Clinical Research Laboratory in Dentistry, Universidade Federal Fluminense, Niteroi 24020-140, Brazil; 9 Private Practice, 24121 Bergamo, Italy; m.montemezzi@libero.it; 10 Orthodontics Department, Universidade Federal Fluminense, Niteroi 24020-140, Brazil

**Keywords:** biomaterial, biocompatibility, cytocompatibility, microtomography

## Abstract

Synthetic biphasic calcium phosphate (BCP) granules and powder are biocompatible biomaterials with a well-known capacity for osteoconduction, presenting very satisfactory clinical and histological results. It remains unanswered if the putty configuration impacts the biological response to the material. In this study, we aimed to compare the cytocompatibility and biocompatibility of nanostructured BCP in the putty configuration (moldable nanostructured calcium phosphate, MnCaP) on the healing of critical-sized bone defects (8 mm) in rat calvaria. Cytocompatibility was determined through the viability of fibroblast cells (V-79) to the extracts of different concentrations of MnCaP. Forty-five Wistar rats were randomly divided into three groups (*n* = 15)—clot, MnCaP, and commercial biphasic calcium phosphate in granules configurations (Nanosynt^®^)—and subdivided into three experimental periods (1, 3, and 6 months). Histological, histomorphometric, and microtomographic analyses allowed the evaluation of newly formed bone, residual biomaterial, and connective tissue. The in vitro evaluation showed that MnCaP was cytocompatible. The histomorphometric results showed that the Nanosynt^®^ group granted the highest new-formed bone values at six months (*p* < 0.05), although the biomaterial volume did not differ between groups. The putty configuration was easier to handle, and both configurations were biocompatible and osteoconductive, presented similar biosorption rates, and preserved the calvaria architecture.

## 1. Introduction

Extensive and localized bone loss caused by periodontal disease, tooth extraction, trauma, or surgical pathological resections compromise bone tissue regeneration [1,2]. Sometimes, the bone repair process is incomplete, and fibrous tissue occupies part of the bone loss area [3,4], which interferes with function and, in most cases, the esthetics and quality of life of affected individuals.

Although autogenous bone is considered to be the gold standard in cases of extensive reconstructions, the disadvantages related to obtaining this, such as morbidity, cost, and its unpredictability, must be considered [5,6,7,8]. A series of studies have been conducted using different biomaterials with osteoconductive and resorbable properties to fill post-extraction cavities to maintain the appropriate alveolar architecture and bone healing favoring further dental implant installation [9,10].

Among the different types of synthetic biomaterials, hydroxyapatite (HA) has been widely studied due to its (1) similarity to the components of the mineral phase of bone tissue, (2) safety regarding the risk of disease transmission, (3) direct union with bone tissue and known biocompatibility, and (4) osteoconductive properties [11,12]. Moreover, HA presents local and systemic safety and when used in its pure form has a low rate of bioabsorption [13], which limits the newly formed bone during bone repair [14].

Beta tricalcium phosphate (β-TCP) has osteoconductive properties and shows a higher bioabsorption rate than HA [15,16]. Biphasic calcium phosphate (BCP) bioceramics consist of a mixture of hydroxyapatite (HA) with β-TCP [17]. The manipulation of the HA/β-TCP ratios may control the bioactivity of the ceramic materials [16,18]. The β-TCP association with HA results in a porous biphasic biomaterial presenting an average degradation rate, promoting a framework for cell migration and the activation of cellular events that occur during bone repair [9].

Biphasic calcium phosphates, which combine 40% β-TCP with 60% HA, may produce a further physiological balance between mechanical support and bone regeneration scaffolds [15].

An in vivo study evaluated the effect of β-TCP and HA ratios on osteoconductivity and concluded that a 20:80 ratio promoted more bone formation compared to the 80:20, 70:30, and 30:70 ratios [16]. Currently, BCP bioceramics represent an alternative or additive to autogenous bone for orthopedic and dental applications [19]. A previous clinical study of alveolar bone preservation compared two biphasic calcium phosphates with different ratios of 60:40 and 78:22, finding that both were biocompatible and osteoconductive. However, the 60:40 ratio (HA/β-TCP) showed the greatest amount of newly formed bone, less connective tissue and less remaining biomaterials after 6 months compared with the other groups [20].

Tridimensional polymeric scaffolds made of composites of PLGA (polylactide-co-glycolide acid), PLA (poly-lactide acid), or PGA (poly-glycolide acid) copolymers associated to biphasic calcium phosphate have as their advantages the improvement of mechanical properties; control of the degradation rate, mainly related to their chemical composition, molecular weight and crystallinity; accelerated hydroxyapatite degradation, releasing calcium and phosphate ions which contribute to novel bone formation; and the pH control of tissues surrounding the implantation site, by buffering the effects of the alkaline degradation products of hydroxyapatite over glycolic and lactic acids released by the polymers. Through their manufacturing process, the modulation of the chemical composition and structural configuration of these polymers allows the control of characteristics which are crucial to bone tissue engineering: high porosity and pore interconnectivity, adequate pore size, biocompatibility, and a degradation rate compatible with bone regeneration [21,22,23].

The forms of BCP available in the market are powder or granules that are not very easy to handle and that frequently do not fit in a bone defect, with some granules being spread out of the surgical area. Moreover, these forms are not possible to mold according to the need of the defect, which would allow the architecture of the bone loss area to be regained. In fact, the putty configurations available for bone substitutes are composites of xenogeneic porcine and equine, bioactive glass, PLGA, BCP with hydrogel, and BCP with polyethylene glycol. It is well known that the granule size, shape, porosity, and crystallinity have important influences on tissue reaction [24,25,26], but it is not known to date if the biomaterial’s configuration can impact the biological response.

Descriptive histological and histomorphometric analyses are the most used and reliable methods for the qualitative and quantitative investigation of bone repair induced by biomaterials. Through the light microscopy image analysis protocol, it is possible to quantify structures such as newly formed bone, connective tissue, and the presence of residual biomaterial. However, these analyses are limited as they do not allow a three-dimensional assessment of the interest area [27,28].

Computed microtomography (micro-CT) is a technique used for the volumetric reconstruction of CT scans of a given sample, thus enabling the tridimensional analysis and a more accurate visualization, evaluation, and description of bone mass. Furthermore, micro-CT is a non-destructive method and, after the scan, the sample can be passed to further analysis [29,30].

Considering the need for a biocompatible and osteoconductive biomaterial for easier handling (putty configuration), in this study, we aim to compare the efficacy of a nanostructured calcium phosphate (MnCaP) on the healing of critical size bone defects (8 mm) in rat calvaria compared to a non-moldable commercial calcium phosphate.

## 2. Materials and Methods

### 2.1. Biomaterial Composition

The composition of the studied biomaterial was previously published in [20]. The percentage of HA and b-TCP phases were shown to be 60.28% and 39.72%, respectively.

### 2.2. Physico-Chemical Characterization of Biomaterials

The crystalline mineral phases present in the samples and the proportion of the hydroxyapatite and β-tricalcium phosphate phases were examined by X-ray diffraction (XRD). A Zeiss HZG4 diffractometer operating at 30 kV and 15 mA with CuKα radiation (λ = 1.542 Å) showed the XRD patterns collected in the 2θ range of 10°–100° with a step of 0.02° points per second. The samples were placed onto the slide in a powdered form for later analysis in the apparatus.

Fourier-transform infrared spectroscopy (FTIR-IR) with a deuterated triglycine sulfate (DTGS) KBr detector (Prestige 21, Shimadzu Corporation, Kyoto, Japan) provided the vibrational modes of phosphate and hydroxyl groups in the samples with 1% KBr in the median infrared range (4000–400 cm^−1^).

Scanning electron microscopy (SEM) showed the different biomaterials morphologies and topography at magnifications of 500× and 5000× (SEM JEOL JSM 5310, Tokyo, Japan).

### 2.3. In Vitro Assay

Fibroblasts from Chinese hamster lungs (V-79) were cultured in Dulbecco’s Modified Eagle’s Medium (DEMEM) (Cultilab, Brazil) medium supplemented with 10% fetal bovine serum (FBS) and antibiotics (10,000 UI/mL penicillin and 10 mg/mL streptomycin) (culture medium) in 96-well-plate subcultures and kept in an incubator at 37 °C for 48 h for the formation of the cell monolayer. Fibroblast cells were chosen for the cytotoxicity evaluation because, during bone repair, the largest number of cells in contact with the biomaterial in a critical size defect are the connective tissue cells, predominantly fibroblasts; therefore, we focused on this cellular type. After this period, the culture medium was replaced by a negative control (C−)according to the culture medium; for extraction control (EC), the culture medium was kept in an incubator at 37 °C in a humidified atmosphere containing 5% CO_2_ for 24 h; for the positive control (C+), the extraction of zinc dibutyldithiocarbamate (ZDBC) or zinc diethyldithiocarbamate (ZDEC) products in the culture medium was performed, and the test material (MnCaP) was investigated at different concentrations of 100%, 50%, 25%, and 12.5% in the extraction medium incubator at 37 °C in a humidified atmosphere containing 5% CO_2_ for 24 h. According to ISO 10993-5/2009, after an incubation period of 24 h, it is possible to determine cytotoxic effects through qualitative and quantitative evaluation; a reduction of cell viability by more than 30% is considered a cytotoxic effect. At the end of the exposure period, the cells were washed with phosphate-buffered saline (PBS). Cellular viability was measured in quintuplicate with a kit (In Cytotox, Xenometrix, Allschwil, Switzerland) through the analysis of mitochondrial activity the reduction of 2, 3-bis (2-methoxy-4-nitro-5-sulfophenyl)- 2H- tetrazolium-5-Carboxanilide) (XTT) to formazan. The absorbance reading of the samples was carried out at a wavelength of 570 nm. According to this protocol (ISO 10993-5/2009), samples that presented a reduction in cell viability greater than 30% to the negative control were considered cytotoxic. The in vitro cytotoxicity test was not performed for the Nanosynt^®^ because this biomaterial is already commercially available and safe for human use [20].

### 2.4. In Vivo Analysis

#### 2.4.1. Ethical Considerations

The animal breeding and procedures followed the conventional guidelines of the NIH Guide for the Care and Use of Laboratory Animals, the Brazilian Directive for the Care and Use of Animals for Scientific and Didactic Purposes—DBCA, and the Euthanasia Practice Guidelines of the CONCEA (Brazilian National Council for the Control of Animal Experimentation). The Ethics Committee of Animal Use from Fluminense Federal University (CEUA/UFF) approved the research protocol of this work (protocol number 874). The report of the present study considered the Animal Research: Reporting of In Vivo Experiments (ARRIVE)guidelines concerning the relevant items [31] supplemented by Planning Research and Experimental Procedures on Animals: Recommendations for Excellence (PREPARE) [32].

#### 2.4.2. Animal Characterization and Location

The Laboratory Animal Center (NAL) of the Fluminense Federal University, Niteroi, provided the 45 male 3 month-old Wistar rats, weighing from 300 to 350 g. Isolators contained a maximum of two animals each throughout the experimental periods, with pelleted food and water freely available. A senior veterinarian monitored the nutritional parameters, animal care and well-being, and pre and post-operative fasting.

#### 2.4.3. Anesthesia and Surgery Procedures

All surgical procedures occurred under general intraperitoneal anesthesia (100 mg/kg of ketamine IM, Virbac^®^, Veltbrands, São Paulo, Brazil, 10 mg/kg of xylazine, Sedazine^®^, Fort Dodge, Rio de Janeiro, RJ, Brazil, and 5 mg/kg of Midazolam, Eurofarma, Rio de Janeiro, RJ, Brazil). Food restriction started six hours before the surgery, but not for water; at the time of surgery, rats were weighed on a precision digital scale. After observing the absence of pain reflexes due to antisepsis in the rat calvaria, a semilunar incision was made over the calvaria, detaching the periosteum. After the exposure of the calvaria, an 8 mm diameter trephine drill made a critical-size surgical defect. Nanostructured calcium phosphate in putty configuration (MnCaP, Dentscare Ltda, Joinvile, SC, Brazil), and Nanosynt^®^ granules (Commercial nanostructured calcium phosphate, Dentscare Ltda, Joinvile, SC, Brazil) filled the bone defect only to the level of the surrounding bone without packing and was gently placed in the defects without displacing the dura. In the sham group, after the same procedures (incision, detachment, and trephining of the calvaria), the blood clot filled the bone defect. The skin was repositioned and sutured with 5.0 Nylon (Technofio, Permed, Mafra, SC, Brazil) to protect the surgical bed. After the surgery was performed by an expert surgeon, the rats were returned to the mini-isolators for anesthetic recovery, with feed and water freely available. Daily evaluation of the animals permitted the registration of any postoperative complications. The animals received an anti-inflammatory drug intramuscularly (1 mL/kg, Maxicam^®^, Ourofino pet—Osasco, São Paulo, SP, Brazil) after surgery and two more doses on subsequent days. Rats were euthanized with a lethal dose of general anesthetic 1, 3, and 6 months after surgery to collect the samples.

#### 2.4.4. Image Acquisition by Micro-CT and 3D Segmentation

One sample from each experimental group and from the 3 months period was fixed in 4% buffered formaldehyde (phosphate buffer, pH 7.2) for 48 h. The samples were scanned in a micro-CT unit (SkyScan 1174, Bruker-microCT, Kontich, Belgium) using pre-selected parameters of 70 kV, 114 mA, and an isotropic pixel size of 14.16 µm. The digitization was done with 360° of rotation around the axis, with an exposure time of 7000 ms per projection and a 0.5° pass rotation step, associated with a camera device. The X-ray was filtered with a 1 mm aluminum filter, removing low-energy X-ray photons in order to reduce beam hardening effects [33], thus acquiring better image quality. A flat field correction before the reading procedures corrected the variations in the camera’s pixel sensitivity. The images were reconstructed using the SkyScan’s volumetric NRecon software v.1.6.3 (Brucker-microCT, Kontich, Belgium) with a beam hardening correction of 40% and artifact correction of 10, resulting in the acquisition of 700–800 transversal projections by acquisition in bitmap format. The DataViewer^®^ program was used for the 2D visualization and evaluation (linear measurements) of the coronal, transaxial, and sagittal axes for the evaluation of the presence of newly formed bone, biomaterial, and connective tissue.

#### 2.4.5. Segmentation Protocol

Thermo Scientific Avizo 9.5 software (Huston, TX, USA) was used to filter, segment, and quantify all phases present in the image: new bone, biomaterial, connective tissue (CT), and background. Before segmentation, a non-local means filter was applied in order to reduce the reconstruction artifacts and facilitate targeting [34]. The attenuation of the X-ray beam when passed through the sample, depending on the density of each sample structure, resulted in differentiation in the grayscale images. Thus, we obtained different grayscale ranges for resident bone together with the newly formed bone, the biomaterial, the connective tissue, and the background.

The background was removed with an interactive threshold tool. In the first segmentation step, a quick segmentation was performed by single thresholding to create seeds attributed to each phase present in the image. The previously created seeds were the input for the fine-tuned watershed-based segmentation of the bone and biomaterial phases [35]. A Lasso 3D tool masked the new bone region located in the defect cavity, separating it from the pre-existing bone. CT was segmented using a magic wand tool, masking the corresponding threshold range. After the segmentation, all phases (pre-existing bone, new bone, biomaterial, and connective tissue) were visualized using Thermo Scientific Avizo 9.5.

#### 2.4.6. Histological Processing

The bone blocks were dissected to remove the soft tissue and fixed in 4% buffered formaldehyde (phosphate buffer, pH 7.2) for 48 h. The decalcification of samples was achieved with 10% buffered ethylenediaminetetraacetic acid (EDTA) (Allkimia^®^, a bone demineralizing solution) for two days at room temperature. The histological process for paraffin embedding was conducted to obtain one longitudinal, 5 μm thick section stained with hematoxylin–eosin (HE). All histologic slides were coded according to the experimental groups and periods, and two experienced examiners blindly evaluated the slides.

#### 2.4.7. Histological and Histomorphometric Evaluation

Each slide stained with HE (*n* = 5, one slide per animal) was observed under an optical microscope (OLYMPUS BX43, Tokyo, Japan), and six photomicrographs corresponding to the regions surrounding the implanted biomaterial were captured by scanning without overlapping, using a high-resolution digital camera (OLYMPUS SC100, Tokyo, Japan). The magnification used in the light microscope was 40× for histomorphometric analysis. For general histological evaluation, magnifications if 10× and 40× were also used to identify the defect area, inflammatory cell infiltration patterns, reminiscent biomaterial, and newly formed bone. Histomorphometric analysis was done using the Image-Pro Plus^®^ 6.0 imaging software (Media Cybernetics, Silver Spring, MD, USA). Through this program, a grid of 133 points was superimposed on the area under analysis, which allowed the determination of the volume density of the newly formed bone, of the connective tissue, and of the residual biomaterial. The 133 points superimposed on each photomicrograph were considered as 100%, so each point was classified, and the percentage of each parameter was obtained (Adapted from [36]). Quantitative values were stored in the Microsoft Excel^®^ database and were transferred to the Prism^®^ 8.0 software (GraphPad Software, Inc., Irvine, CA, USA) for statistical analysis.

#### 2.4.8. Statistical Analysis

The percentage of each analyzed structure was submitted to the Rout test (Q = 1%) to exclude outliers. The Shapiro–Wilk normality test (alpha = 0.05) demonstrated that some groups did not pass the normality test. The comparison between the experimental groups for each evaluated experimental time was performed using the Kruskal–Wallis test. The differences between the groups evaluated were determined by Dunn’s multiple comparison test, considering significant differences for *p* < 0.05.

## 3. Results

### 3.1. Biomaterial-Related Characterization

**Figure 1:**
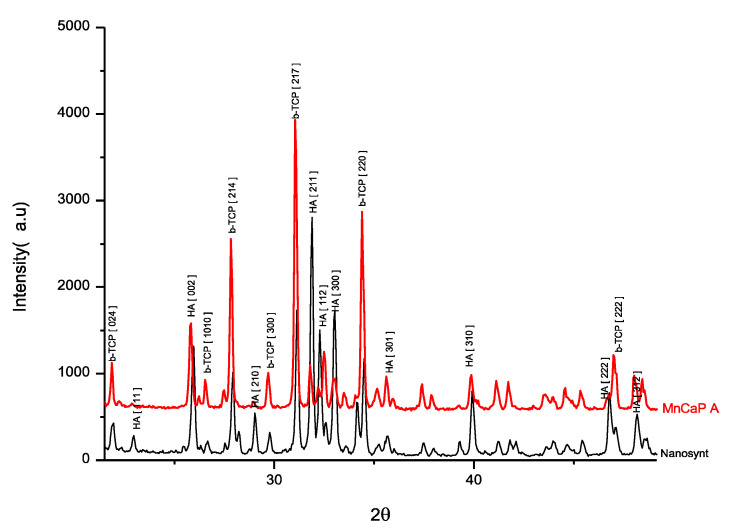
X-ray diffraction pattern (XRD) of MnCaP and Nanosynt^®^ samples, with a typical pattern of biphasic calcium phosphate, with mostly peaks of hydroxyapatite (HA) and beta tricalcium phosphate (β-TCP). MnCaP, red line; and Nanosynt^®^, black line.

The biomaterials showed typical X-ray diffraction (XRD) patterns of biphasic calcium phosphate, with mostly peaks of HA; according to the PCPDFWIN 09.0432 pattern, the β-TCP phase was responsible for the biomaterial dissolution in biological media (Figure 1).

**Figure 2:**
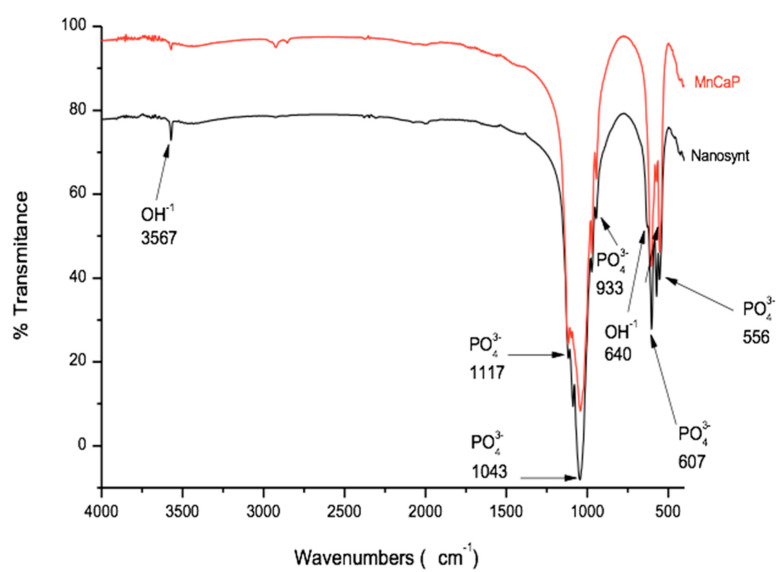
Fourier transform infrared (FTIR) spectrums. The FTIR spectrum showed vibrational modes typical of calcium phosphate with (PO_4_)^3−^ at 556 cm^−1^, 607 cm^−1^, 933 cm^−1^, 1.043 cm^−1^, and 1117 cm^−1^ and vibrational modes of hydroxyl (OH^−^) at 640 cm^−1^ and 3567 cm^−1^ bands with a well-formed HA structure. MnCaP, red line; and Nanosynt^®^, black line.

The FTIR spectrum showed vibrational modes of typical calcium phosphate with (PO_4_)^3−^ at 556 cm^−1^, 607 cm^−1^, 933 cm^−1^, 1.043 cm^−1^, and 1117 cm^−1^, and vibrational modes of hydroxyl (OH^−^) at 640 cm^−1^ and 3567 cm^−1^ bands with a well-formed HA structure (Figure 2), confirming the XRD results.

**Figure 3:**
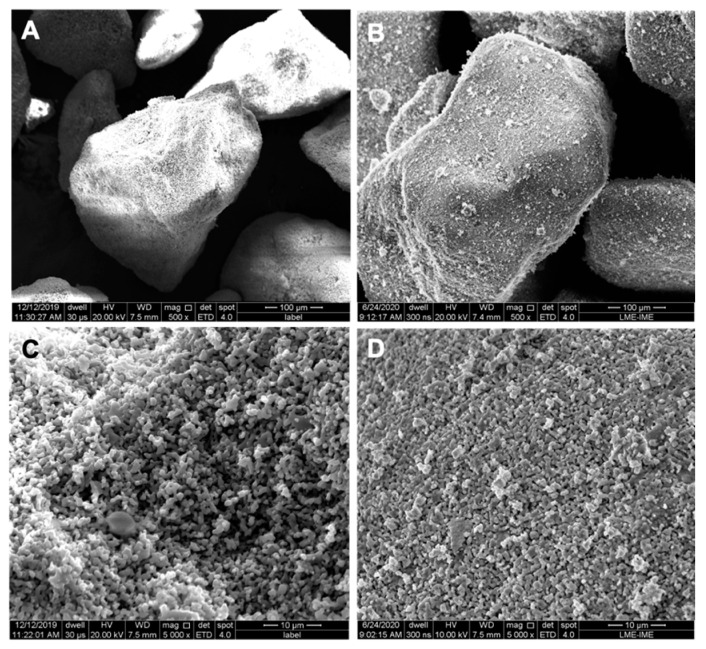
Scanning Electron Microscopy (SEM) micrographs. MnCaP (**A**,**C**) and Nanosynt^®^ (**B**,**D**). Magnification: (**A**,**B**): 500×; (**C**,**D**): 5000×.

SEM analyses with a sub-micron resolution were conducted in both biomaterials, as illustrated in Figure 3. The biomaterial MnCaP appeared to have rough surface around 500 µm (A and C), and was very similar in 500× and 5000× magnifications to the Nanosynt^®^ group (B and D). The 5000× magnification showed an agglomeration of very small particles of around 1 um in diameter for both groups.

### 3.2. Cytocompatibility

**Figure 4:**
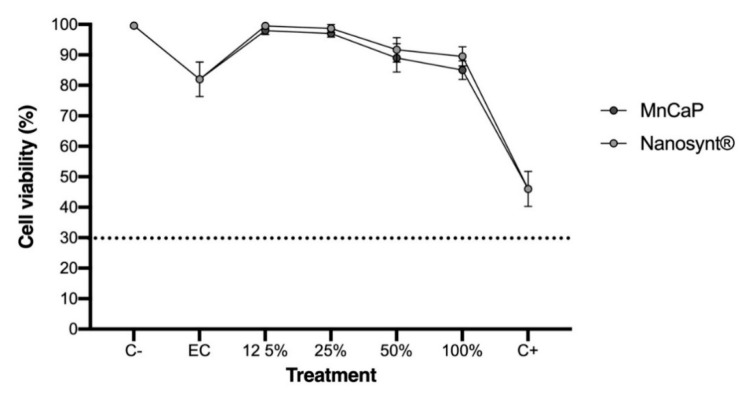
Assay of cytotoxicity in fibroblasts from Chinese hamster lung (V-79). Mitochondrial activity measured by XTT reduction. The results are presented as mean ± confidence interval (*n* = 5). All biomaterial concentrations were cytocompatible, showing a reduction of cell viability less than 30% from the negative control. There was no difference between MnCaP and Nanosynt^®^ groups in the same concentrations. Abbreviations: C−, negative control; EC, extraction control; C+, positive control. XTT: 2, 3-bis (2-methoxy-4-nitro-5-sulfophenyl)- 2H- tetrazolium-5-Carboxanilide).

Figure 4 reveals the expected toxicity for the positive control (C+) relative to the negative control (C−). All biomaterial concentrations were cytocompatible, showing a reduction of cell viability of less than 30% from the negative control.

### 3.3. Descriptive Histology

**Figure 5:**
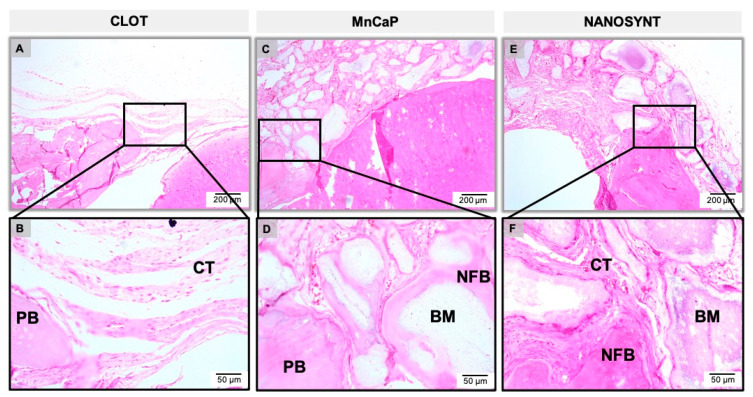
Micrographs of clot, MnCaP, and Nanosynt^®^ groups after 1 month of implantation, respectively. Magnification: 10× (**A**,**C**,**E**) and 40× (**B**,**D**,**F**). The squares indicate the magnified area observed using a 40× objective. Abbreviations: PB, pre-existing bone; BM, biomaterial; NFB, new formed bone; CT, connective tissue. Stain: HE: hematoxylin–eosin.

**Figure 6:**
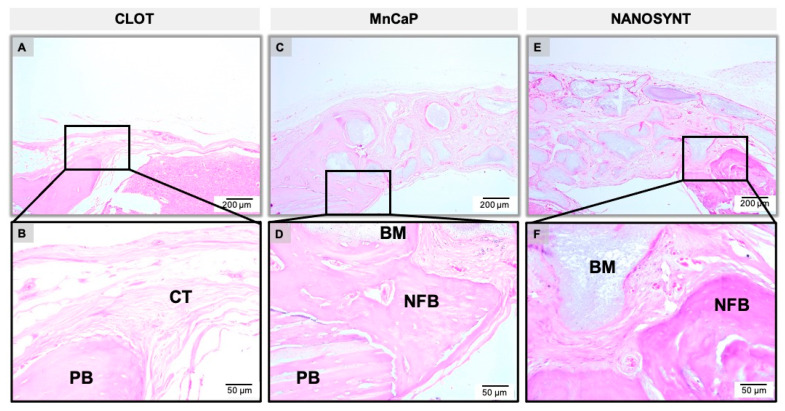
Micrographs of clot, MnCaP, and Nanosynt^®^ groups after 3 months of implantation, respectively. Magnification: 10× (**A**,**C**,**E**) and 40× (**B**,**D**,**F**). The squares indicate the magnified area observed using a 40× objective. Stain: HE.

**Figure 7:**
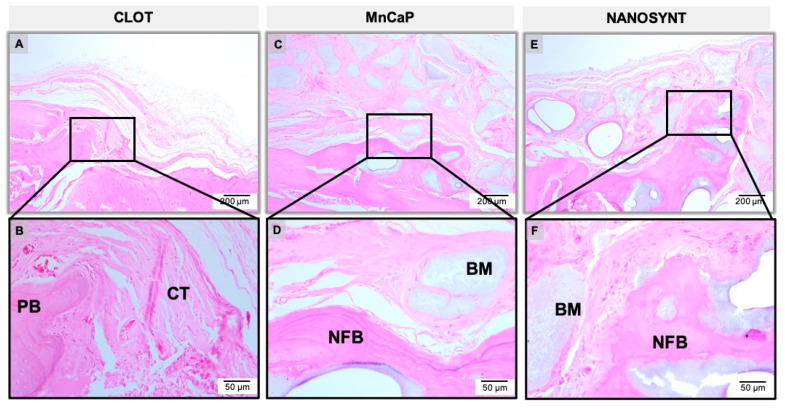
Micrographs of clot, MnCaP, and Nanosynt^®^ groups after 6 months of implantation, respectively. Magnification: 10× (**A**,**C**,**E**) and 40× (**B**,**D**,**F**). The squares indicate the magnified area observed using a 40× objective. Stain: HE.

The results of the descriptive histological analysis were divided according to experimental groups and subdivided according to the experimental periods (Figure 5, Figure 6 and Figure 7).

#### 3.3.1. One Month

The clot group, which was filled exclusively with blood, showed abundant loose connective tissue, an absence of newly formed bone (NFB) in the center of the defect, and minimal presence of NFB on the margins of the defect one month after surgery (Figure 5A,B). In the MnCaP group, a large number of particles of varying sizes and shapes of biomaterial interspersed with connective tissue was observed (Figure 5C,D). Multinucleated giant cells populated the periphery of the biomaterial (not shown). In the Nanosynt^®^ group, there was a large amount of biomaterial of varying size and shape, with the presence of multinucleated giant cells on the periphery of the biomaterial interspersed by connective tissue (not shown). A small amount of NFB was observed in the periphery of the defect and between the particles of the biomaterial (Figure 5E,F). There were no differences observed in inflammatory cell populations between MnCaP and Nanosynt^®^.

#### 3.3.2. Three Months

After three months, in the sample only containing clots, a thin band of fibrous connective tissue was present, with no NFB and scarce inflammatory infiltrate (Figure 6A,B). In the MnCaP group, the presence of a large amount of biomaterial of different sizes and shapes persisted. It was also possible to observe some multinucleated giant cells involving particles of the biomaterial. The presence of NFB in the periphery of the defect towards the center and sometimes involving the biomaterial particles was evidenced (Figure 6C,D). In the Nanosynt^®^ group, the defect was filled by a large number of biomaterial particles interspersed with fibrous connective tissue containing multinucleated giant cells. A small NFB volume was observed in the margins of the defect (Figure 6E,F).

#### 3.3.3. Six Months

In the defect filled with the blood clot, a thin band of fibrous and loose connective tissue was observed. In addition, this period showed the absence of inflammatory infiltrate and some NFB in the periphery of the bone defect (Figure 7A,B). The MnCaP group presented a large amount of biomaterial interspersed with fibrous connective tissue, and areas of NFB on the periphery of the defects, sometimes involving the particles of the biomaterial. The presence of multinucleated giant cells was scarce at this period (Figure 7C,D). The Nanosynt^®^ group showed a large amount of biomaterial of different sizes and shapes, interspersed with fibrous connective tissue, the presence of NFB from the periphery towards the center, sometimes involving particles of biomaterial, and the presence of giant cells involving biomaterial (Figure 7E,F) Both biomaterials presented higher amounts of NFB compared to the previous period.

**Figure 8:**
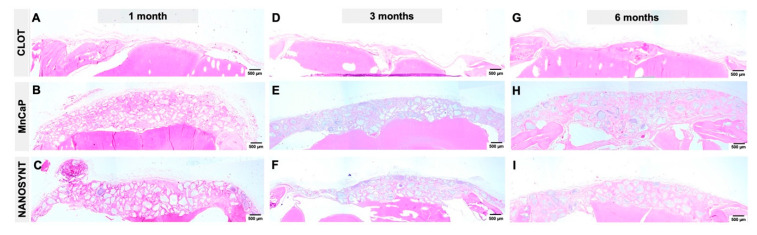
Representative photomicrographs of biological response to biomaterials implanted in calvaria defects after experimental periods. Histological reconstruction of the coronal sections from the clot (**A**,**D**,**G**), MnCaP (**B**,**E**,**H**) and Nanosynt^®^ (**C**,**F**,**I**) groups. The photomicrographs are displayed in three columns that represent the experimental periods at 1, 3, and 6 months post-surgery. HE staining. Scale bar: 500 µm.

The histological reconstruction of the rat calvaria in different groups is shown in Figure 8. The reconstruction is shown according to group—the clot (Figure 8A,D,G), MnCaP (Figure 8B,E,H), and Nanosynt^®^ (Figure 8C,F,I) groups—and according to the experimental periods: 1 month (Figure 8A–C), 3 months (Figure 8D–F) and 6 months (Figure 8G–I).

### 3.4. Histomorphometric Results

**Figure 9:**
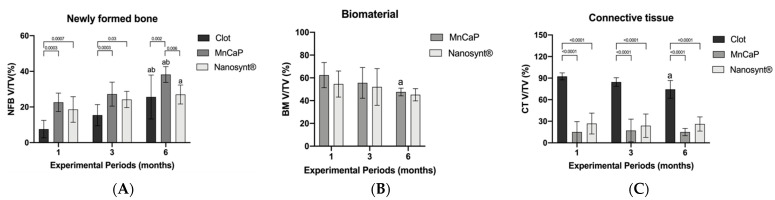
Statistical results for newly formed bone (**A**), biomaterial (**B**), and connective tissue (**C**). (**A**). Newly formed bone volume/total volume (NFB V/TV (%)). Rates of NFB V/TV (%) of clot, MnCaP and Nanosynt^®^ groups after 1, 3 and 6 months of implantation (*n* = 5). The values are presented as means ± confidence intervals. The differences between the groups in the same experimental period (represented by horizontal bars) and in the same group at different experimental periods (represented by letters) were determined by ANOVA and Tukey’s post-test (*p* < 0.05). (a) represents significant differences compared to the same group at 1 month; (b) represents significant differences compared to the same group at 3 months. (**B**). Biomaterial volume/total volume (BM V/TV (%)). Rates of BM V/TV (%) of MnCaP and Nanosynt^®^ groups after 1, 3 and 6 months of implantation (*n* = 5). The values are presented as means ± confidence intervals. The differences between the groups in the same experimental period were determined by the Student’s *t*-test. The differences between the same group at different experimental periods (represented by letter) were determined by ANOVA and Tukey’s post-test (*p* < 0.05). (a) represents significant differences compared to the same group at 1 month. (**C**). Connective tissue volume/total volume (CT V/TV (%)). Rates of CT V/TV (%) of clot, MnCaP and Nanosynt^®^ groups after 1, 3 and 6 months of implantation (*n* = 5). The values are presented as means ± confidence intervals. The differences between the groups in the same experimental period (represented by horizontal bars) and same group in different experimental periods (represented by letters) were determined by ANOVA and Tukey’s post-test (*p* < 0.05). (a) represents significant differences compared to the same group at 1 month.

Figure 9 summarizes the main results related to the percentage of NFB, biomaterial, and connective tissue volume density, respectively.

After 1 month, higher values of NFB were observed in MnCaP (22.6 ± 4.15) and Nanosynt^®^ (18.6 ± 5.7) groups compared to the clot group (7.6 ± 3.9). However, there were no differences between the biomaterial groups. After 3 months, this profile was maintained, with similar values presented by the biomaterials groups and a reduction shown in the clot group (clot 15.4 ± 4.7; MnCaP 27.2 ± 5.4; 24.2 ± 3.7. At 6 months post-surgery, the MnCaP (38.2 ± 3.5) group presented the highest NFB compared to clot (25.6 ± 9.9) and Nanosynt^®^ (27 ± 4.3) groups (*p* < 0.05) (Figure 9).

**Figure 10:**
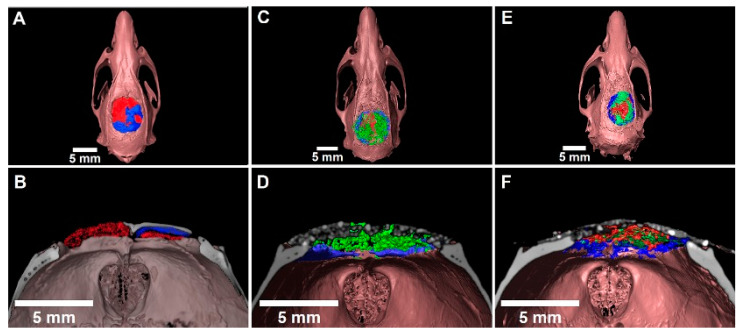
Volume rendering of defects in calvaria obtained from µCT sections. Through histological correlation, the following were segmented: (**A**,**B**). Clot group with new formed bone towards the edges of the defect in the center; (**C**,**D**). MnCaP with new formed bone, and connective tissue in the center of the defect; (**E**,**F**). Biomaterial with connective tissue, and new formed bone. New formed bone: blue; connective tissue: red; and biomaterial: green.

The volume of remaining biomaterial in MnCaP and Nanosynt^®^ groups presented no differences after the experimental periods: 1 month (MnCaP ± 8.8; Nanosynt^®^ 54.6 ± 9.2); 3 months (55.6 ± 10.8; 52 ± 12.9); and 6 months 47.6 ± 2.7; 45.2 ± 4.3). The volume of MnCaP presented a reduction after 6 months compared to the first experimental period. The volume of Nanosynt^®^ remained stable throughout the experimental periods (Figure 10).

At the connective tissue parameter, the clot group showed a higher density of connective tissue volume in all experimental periods. There were no differences observed between biomaterials groups throughout experimental periods (*p* < 0.05) (Figure 9).

### 3.5. Three-Dimensional Evaluation by Micro-CT

The micro-CT analysis was conducted at three months after surgery in one animal randomly picked from each experimental group. In the clot group, the new formed bone was observed in a direction to the center from the resident bone in the border. The blue color represents the segmentation of the newly formed bone structure in the calvaria defect (Figure 9A), presenting a thin bone layer in a coronal section (Figure 9B).

In the sample containing MnCaP, the presence of radiolucent spots compatible with biomaterial inside the defect was observed (green area). At the periphery of the defect, we observed biomaterial surrounded by mineralized tissue with the same density as the resident bone (Blue area) (Figure 10C). In the coronal section (Figure 10D), we observed the new bone formation around the biomaterial.

In the Nanosynt^®^ group, the presence of radiolucent spots compatible with the density of the new formed bone was observed (green area), surrounded by connective tissue (red area) (Figure 10E).

## 4. Discussion

Critical-sized defects in rat calvaria do not regenerate completely and spontaneously during the animal’s lifetime [37,38]. This model is very interesting and challenging due to the poor blood supply at the defect site, the lack of muscles, and the tiny amount of bone marrow as a potential source for stem cells. For a critical-sized calvaria defect of 8 mm, it is not possible to complete the bone regeneration without the use of regenerative therapy, as there is no support structure for the deposition of the bone matrix, so the complete regeneration of the defect is not achieved without osteoconductive materials.

Moreover, the bone quality of rat calvaria is similar to human craniomaxillofacial bone [39]. This model has been used for biomaterial evaluation in previous studies [40,41].

In this study, in all experimental groups, complete bone regeneration was not observed after six months of treatment; these results were also observed in other studies that did not use osteoinductive biomaterials [41,42,43].

Currently, there are different biphasic calcium phosphates available commercially, but all are in the shape of powders or granules [44]. This study used a biphasic calcium phosphate containing a polymer that allowed the material to be moldable during its surgical application. The moldable material allows better handling and adaptation to the bone defect, with the advantage of regaining the height and thickness that are usually lost after tooth extractions.

After surgery, no adverse clinical events occurred that resulted in atypical behavior from the animals. However, it is worthwhile to highlight that the ease of manipulation and usability of the biomaterial were very good, ensuring the quick and safe insertion of the material into the surgical site without spreading out the granules of the materials outside the bone defect. The bone graft-substitute particles were integrated into the pre-existing bone at the margins of the defect, and the absence of the important inflammatory response and scarce giant cells characteristic of foreign body reactions indicates the biocompatibility of all tested materials (Figure 5, Figure 6 and Figure 7).

The histomorphometric results showed that MnCaP and Nanosynt^®^ in all experimental periods did not promote new formed bone in the center area of the defect; this result was also observed in previous studies after the same experimental periods [41,42,43].

Histomorphometric and µCT analyses indicated that the tested materials were not totally bioabsorbable, and some granules maintained their original shape in the experimental conditions of this study. Since the biomaterials shared a similar physical and chemical characteristic and standardized amounts of material were used, the similar behavior between them was not a surprise. The lack of biodegradability could be attributed to the thermic treatment of the biomaterial during synthesis and processing, which was similar to a previous study that also used sintered and moldable TCP and other HA/β-TCP composites in a ratio of 60:40 (HA/β-TCP) [44].

In the study by Schimidln et al. (2011) [45], as the BCP contained 60% HA and 40% β-TCP with a small amount of polymer, the authors stated that the TCP content dissolved into Ca^2+^ and PO_4_^3−^ ions, while HA maintained its structure and was not resorbed. Recent data suggest that the β-TCP content of the BCP material may be gradually substituted by calcium-deficient HA [46] and/or by the bone matrix [47]. Possibly, the same behavior occurred in our study, since it was possible to observe the presence of granules until the six months period.

Osteoconduction refers to bone ingrowth from bone defect margins towards the surface or down into the pores of a biomaterial, which serves as a scaffold or template to guide new bone tissue formation [48]. Osteoconduction is frequently seen after BCP implantation and is dependent on both biological factors for bone repair and the physical and chemical properties of the implanted biomaterial (geometry, porosity, and crystallinity) [24,26]. The histological and uCT analyses done in the present work confirmed the ability of all tested material to act as a scaffold for bone grown from the borders of the defect to the center of the lesion.

The segmentation process is a critical step in image analysis, and we subjectively separated the regions of interest into groups of mineralized and unmined structures. Errors at this stage of the analysis of the micro-CT cuts will lead to misinterpretations of the results. In our study, in addition to microtomography analysis, histomorphometric evaluation was also performed, and in both analyses, it was possible to observe the same bone neoformation pattern.

In this study, we performed micro-CT for all experimental groups at a period of 3 months after surgery in order to understand the intermediary experimental period. The micro-CT allowed us to better understand the bone regeneration of the defect (Figure 9). Bony bridging of the osteotomy site is a clinically important parameter outcome, and it was assessed to quantify the defect healing with histomorphometric evaluation (2D) and with µCT evaluation (3D). No significant differences in biomaterial amounts were detected between the biomaterials tested according to histomorphometric evaluation (Figure 9). In a comparative analysis with the clot group, we can see that the tested biomaterials demonstrated the ability to serve as a biological scaffold for the promotion of bone neoformation, while the clot group did not serve as a bridge (Figure 9). Another important parameter that was observed with micro-CT was the bridge between the two extremities of the defect and bone conduction from the margin of the defect toward the center of the defect. This result corroborates the results of a previous study [40] that used calcium phosphate with and without osteoinductive materials, and the groups without rh BMP were not bridged.

As proposed, the implantation of the biphasic calcium phosphate moldable material was effective at preserving the architecture of rat calvaria, as shown in Figure 9.

Biphasic calcium phosphate ceramics are successfully used for bone substitution in many different clinical situations, such as in the repair of bone defects, bone augmentation in orthopedics, periodontal treatment, or as coatings for metallic implants. Innovative strategies of bone tissue engineering with a moldable BCP-based composite associated with cells or inductive agents as BMPs may eliminate the need for autologous bone grafting procedures.

Similarly to other calcium phosphate-based biomaterials, nanostructured biphasic calcium phosphate in granules and putty configurations were not able to regenerate the critical-sized bone defects in the skull of rats; however, due to their biocompatibility, they favored the bone grown from the border to the center of the lesion in higher levels than in the sham group. Importantly, the putty configuration was shown to improve clinicians’ handling during the bone grafting procedure, and it can also fit different sizes and shapes of grafting sites, preserving the original graft shape.

## 5. Conclusions

Based on these results, and despite the limitations of this study, it can be concluded that both biomaterials studied were shown to be osteoconductors, biocompatible, to present similar biosorption rates, and to preserve the calvaria architecture. The biomaterial in a putty configuration presented easier handling during biomaterial implantation.

## Data Availability

The data presented in this study are available on request from the corresponding author.
